# Perfusion Flow Enhances Viability and Migratory Phenotype in 3D-Cultured Breast Cancer Cells

**DOI:** 10.1007/s10439-021-02727-w

**Published:** 2021-02-04

**Authors:** Alice Pasini, Joseph Lovecchio, Marilisa Cortesi, Chiara Liverani, Chiara Spadazzi, Laura Mercatali, Toni Ibrahim, Emanuele Giordano

**Affiliations:** 1 Laboratory of Cellular and Molecular Engineering “Silvio Cavalcanti”, Department of Electrical, Electronic and Information Engineering “G. Marconi” (DEI), Alma Mater Studiorum – University of Bologna, Cesena, FC Italy; 2 Osteoncology and Rare Tumors Center, Istituto Scientifico Romagnolo per lo Studio e la Cura dei Tumori (IRST) IRCCS, Meldola, Italy; 3 BioEngLab, Health Science and Technology, Interdepartmental Center for Industrial Research (HST-CIRI), Alma Mater Studiorum – University of Bologna, Ozzano Emilia, Italy; 4 Advanced Research Center on Electronic Systems (ARCES), Alma Mater Studiorum – University of Bologna, Bologna, Italy

**Keywords:** 3D hydrogel scaffold, Cell distribution, Cell eccentricity, Cell migration, MDA-MB-231, Perfusion bioreactor systems, Rat tail collagen, Scratch wound healing assay

## Abstract

Conventional 2D cell culture, a traditional tool in pre-clinical studies, can hardly be regarded as a representation of a natural cell microenvironment. In this respect, it might result in altered cellular behaviors. To overcome such a limitation, different approaches have been tested to conduct more representative *in vitro* studies. In particular, the use of 3D cell culture introduces variables, such as cell-cell and cell-extracellular matrix interactions; cell features such as survival, proliferation and migration are consequently influenced. For an example, an enhanced drug resistance and increased invasiveness are shown by cancer cells when cultured in 3D *versus* 2D conventional culture models. In this setting however, non-uniform cell distribution and biological behaviors appear throughout the scaffold, due to reduced diffusion of oxygen and nutrients. Perfusion in bioreactor systems can be used to improve medium transport. In this line of reasoning, this study proposes a breast cancer cell culture model sustained by an integrated approach that couples a 3D environment and a fluid perfusion. This model improves viability and uniformness of cell distribution, while inducing morphological, functional and molecular cancer cell remodeling.

## Introduction

Conventional 2D cell culture is a reproducible, low cost, experimental model, easily used for multiple purposes, spanning from characterization of normal or disease pathophysiology to drug screening pre-clinical studies. Although this approach has granted a wealth of research data, some of its features – such as the absence of 3D extracellular matrix (ECM)—limit its biomimicry.^17^,^26^ Indeed, glass or standard plastic 2D supports, characterized by stiffness in the GPa magnitude order, do not appear representative of most biological tissues.^27^ To overcome such limitations, different approaches and biomaterials have been tested to conduct *in vitro* studies using 3D cell culture models.^5^,^46^ Culturing cells in a 3D setting introduces variables, such as cell-cell and cell-ECM interactions, that can better mimic pathophysiological conditions, affecting cell survival, proliferation and migration. Indeed, cancer cells display enhanced drug resistance and increased invasiveness when cultured in 3D compared to 2D conventional models.^3^,^4^,^10^,^31^,^34^,^38^,^39^

Culturing cells in a 3D platform increases the relevance of the role of diffusive exchanges. In this respect, culture conditions largely differ from 2D culture, where all the cells have uniform access to oxygen, nutrients and soluble molecules, such as drugs or cytokines. Indeed, multiple studies^13^,^19^,^33^ have shown a non-uniform cell distribution and biological behavior within a 3D scaffold, that often suffers impeded diffusion of oxygen and nutrients inside its structure. Perfusion bioreactor systems have been used to improve medium transport within a scaffold that can contribute to reproduce physiological *in vivo* conditions, as suggested by many examples published by our or other research groups.^11^,^25^,^28^,^36^^–^37,40

The aim of this study was to evaluate viability and cell distribution, together with morphological, functional and molecular cancer cell phenotype in a MDA-MB-231 breast cancer cell culture model sustained by an integrated approach that couples 3D cell culture and perfusion. Triple-negative breast cancer, which does not express estrogen and progesterone receptor, and does not have human epidermal growth factor receptor 2 amplification, is an aggressive neoplastic form with limited treatment options. Many studies on potentially active agents for this particular type of breast cancer have been conducted using MDA-MB-231 cells, which are also recognized as an appropriate model for bone metastasis research.^9^,^29^,^47^ Cells were grown in 3D collagen hydrogels both in standard static and dynamic conditions. Perfusion was applied through a custom-made bioreactor^43^ and its effect on cell growth and proliferation was evaluated, together with the cell distribution within the scaffold, and their morphological, functional and molecular phenotype. Throughout this analysis, standard *in vitro* techniques were coupled with *ad-hoc* image analysis approaches to provide accurate results quantification.

## Materials and Methods

### Perfusion Bioreactor

**Figure 1:**
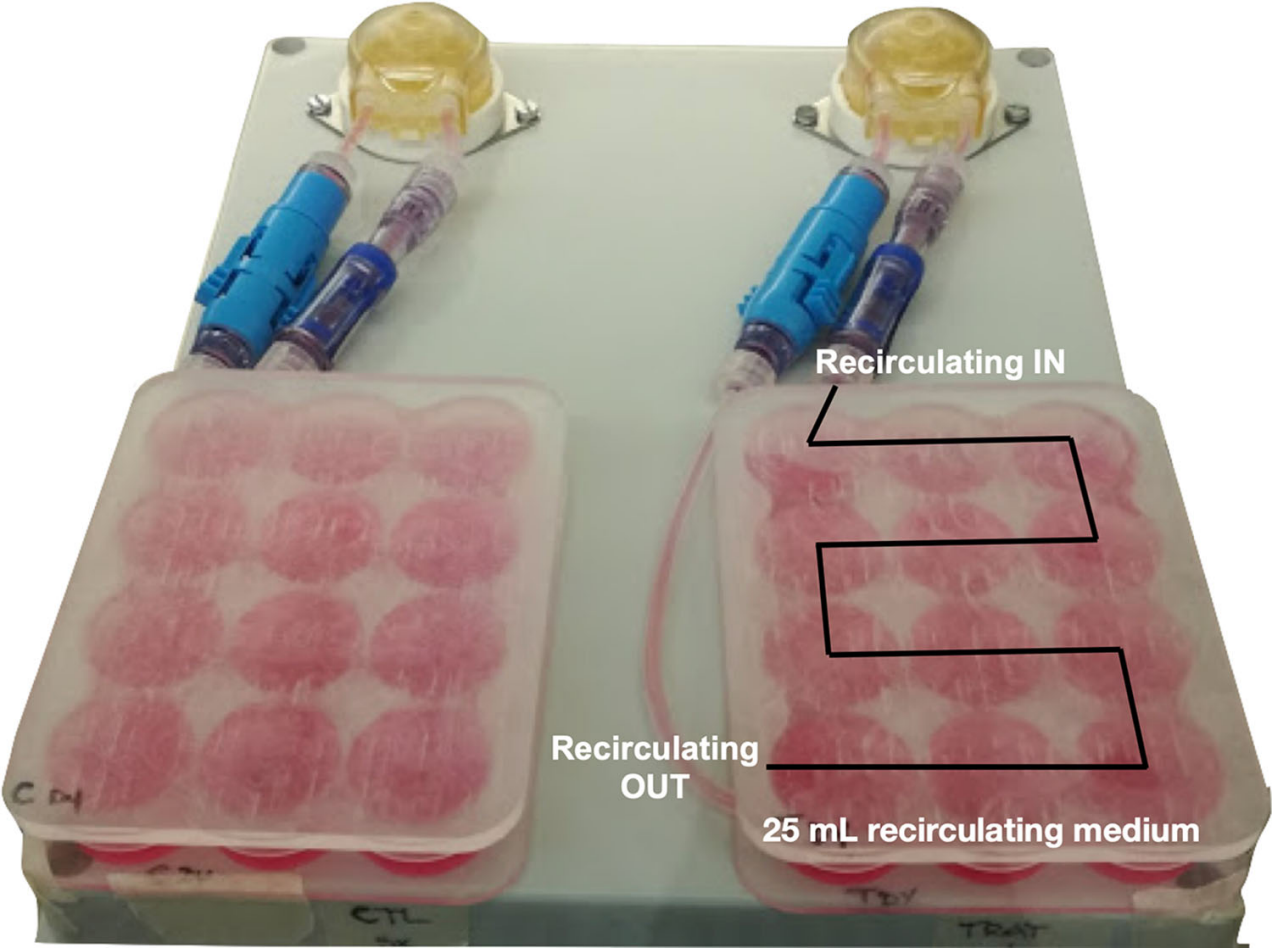
Schematic representation of perfusion bioreactor system.

A perfusion bioreactor system^43^ was used to generate a continuous perfusion flow (Fig. 1) to sustain the cell culture. The device was realised with a unibody plastic case (190L, 240W, and 90H mm in dimension) and includes two autoclavable peristaltic pumps administering a perfusion flow rate of 1 mL/min. Two 12-well poly(methyl methacrylate) (PMMA) custom-milled culture chambers are used to operate independently to allow comparison of two different culture conditions. In particular, a series of holes (2Ø mm) were drilled between adjacent wells to allow medium flow-through. All the material are approved for biomedical application.

### Cell Culture

Triple-negative human breast cancer cell line MDA-MB-231 obtained from the America Type Culture Collection (Rockville, Maryland, USA) was cultured in Dulbecco’s Modified Eagle’s Medium (DMEM) High Glucose (Euroclone S.p.A., Pero, MI, Italia) supplemented with 10% di Fetal Bovine Serum (FBS), 2 mM di L-Glutamine, 100 U/mL Penicillin and 100 mg/ml Streptomycin, at 37 °C in a humidified 5% CO_2_ incubator.

### Collagen Hydrogel Preparation

Collagen hydrogels (10H and 5Ø mm in dimension) were prepared as previously described.^45^ Briefly, RatCol^®^ Rat Tail Collagen for 3D Hydrogels (Advanced BioMatrix, Carlsbad, CA, USA) and its neutralization solution were mixed in the proportion 9:1 keeping the solution in ice to prevent polymerization. Then cell suspension containing 10^5^ cells was added to a final volume of 300 µL. Hydrogels were let polymerize in 96 well plates for 1 h at 37 °C and then transferred in 2 mL medium/scaffold (well) in the 12-well custom PMMA culture chambers. These were then connected to the bioreactor system operating for up to 7 days, supplying fresh medium three times/week.

### Cell Counting

To quantify cell viability, scaffolds were dissolved with 0.25% collagenase (C0130, Sigma-Aldrich, Milano, Italy) in 600 µL of DMEM without FBS during 40 min at 37 °C at indicated time points (2, 4 and 7 days of static or dynamic culture conditions). Cell pellets were then suspended in fresh medium and cells were counted using a Burker chamber, diluting cells 1:2 with Erythrosin B (198269, Sigma-Aldrich) to stain dead cells. Mean values ± SEM were reported and statistical analysis was performed using the Mann-Whitney test by GraphPad Prism 6^®^ software.

### Live/Dead Assay and Cell Viability Quantification

Scaffolds with MDA-MB-231 cells cultured for 7 days in static vs. dynamic condition were processed with the LIVE/DEAD^®^ Viability/Citotoxicity Kit (LIVE/DEAD^®^ Viability/Citotoxicity Kit (MP03224, Molecular Probes™, Eugene, OR, USA) following manufacturer’s instructions. Briefly, hydrogels were washed with PBS and incubated for 45 min at room temperature (rt) in 300 µL of staining solution, which contains 2 μM Calcein AM and 4μM Ethidium homodimer-1 diluted in PBS. Scaffolds were cut in half along the transversal plane using a scalpel. Images were acquired as described previously^29^ using a N-SIM E laser confocal microscope (Nikon Corporation).

Quantitative analysis of cell viability was performed processing images using the ImageJ software in order to calculate the green/red (LIVE/DEAD^®^) area referred to the specific spots following the maximum entropy threshold-based image segmentation method. The total area (A) covered by specific spots of interest in the total scaffold slice surface was calculated using the following formula:

$$A = np \cdot pd^{2}$$

where *np* is the number of pixels detected and *pd* is each pixel’s dimension.

Mean values ± SD were reported and statistical analysis was performed using the Mann-Whitney test by GraphPad Prism 6^®^ software.

### DAPI/Phalloidin Staining

Hydrogels cultured for 7 days in static and dynamic conditions were processed as in Ref.^48^. Briefly, scaffolds were washed 3 times with PBS and cells were fixed using 4% paraformaldehyde (Electron Microscopy Sciences, PA, USA) for 20 min at rt. Membrane permeabilization was obtained with an incubation step in 0.1% Triton X-100 for 5 min. Phalloidin (Life Technologies, CA, USA), diluted 1:40 in PBS, was used to stain F-actin through incubation for 20 min in the dark at rt. Scaffolds were then washed 3 times with PBS and nuclei were counterstained with DAPI. Scaffolds were cut in half along the transversal plane using a scalpel. Images were acquired using the N-SIM E laser confocal microscope.

### Quantification of Spatial Distribution of Cells Within the Scaffold

To study how perfusion influences cell occupancy within the scaffold, we analyzed images of DAPI/Phalloidin-stained cells acquired with a confocal microscope from different regions (core, edge) of sections of the 3D scaffold.

The blue channel was segmented using the Otsu’s algorithm^42^ (threshold set to 0) followed by a morphological opening realized with a diamond kernel of height 6 pixels. This procedure was shown to effectively identify the nuclei of the cells, thus allowing for the quantification of the number of cells in each image. The non-parametric Kruskal–Wallis test was used to determine the statistical difference among the tested conditions.

### Evaluation of Cell Morphology

The study of the influence of perfusion on cell shape relied on images of DAPI/Phalloidin-stained cells acquired with a confocal microscope in random points on a scaffold section.

For this analysis the fluorescent signal tagging the cytoskeleton (Phalloidin) was used. Images were binarized with an adaptive threshold with high sensitivity (0.9 in the range [0,1]). Successively objects with area below 50 pixels were removed and a hole filling procedure, aimed at improving the faithfulness of cell shape, was applied. Finally, the eccentricity distribution was computed. This is equivalent to the aspect ratio used in Ref.^16^ to quantify actin fiber alignment and was computed for each cell using the following equation, where a and b represent the semi-major and semi-minor axes of each cell, respectively.

$$E = \sqrt {1 - \frac{{b^{2} }}{{a^{2} }}}$$

Statistical analysis was conducted under the Kolmogorov-Smirnov 2 samples test.

### Wound Healing Assay

MDA-MB-231 cells were cultured in 3D scaffolds in static vs. dynamic conditions for 7 days. Collagen scaffold were digested using 0.1% collagenase in 600 µl of serum-free medium for 40 min at 37 °C. Cells extracted from the scaffolds were then washed with fresh media and counted. 2 × 10^5^ cells/cm^2^ were seeded in standard 12 well plates and let them growth until confluency for 48 h. A 200 µL tip was used to create the wound and two images of the wound specific to the right and left side of a orthogonal row, previously created with a scalpel on the external bottom of the well, were captured at 0, 2, 4, 6, 8, 24 h post wound, as described in Ref. 44. To identify wound area (free cell area) images were analyzed by AIM software.^15^ Wound area specific to each time point was normalized to the relative maximum cell free area corresponding to the one identified at time 0 h and expressed as percentages. When the wound was completely repopulated of cells, cell free area was set to 0.01%. Data are presented as mean value ± SEM of multiple images per condition (static vs. dynamic cell culture) relative to multiple wells/scaffolds. Experimental points were fitted with an exponential regression curve using GraphPad Prism 6^®^. Statistical analysis was performed using the Mann-Whitney test by GraphPad Prism 6^®^ software.

### Gene Expression Analysis

MDA-MB-231 cells were collected from scaffolds by collagenase digestion after 4 days of culture under static or dynamic condition. Total RNA was extracted using the NucleoSpin^®^RNA (Macherey Nagel, Düren, Germany) following the manufacturer’s instructions. Five hundred nanograms of RNA were reverse-transcribed using the iScript cDNA Synthesis Kit (BioRad, CA, USA). Real-Time PCR was performed on the 7500 Real-Time PCR System (Applied Biosystems, CA, USA) using either TaqMan or SYBR green chemistry (Applied Biosystems) according to the specific target gene assay. The following markers were analyzed: Lysyl Oxidase (*LOX*), matrix metalloproteinase-2 (*MMP2*); matrix metalloproteinase-3 (*MMP3*) and matrix metalloproteinase-9 (*MMP9*), Ras homolog family member A (*RHOA*), Vimentin (*VIM*). The stably expressed endogenous B-actin (*ACTB*) and Hypoxanthine Phosphoribosyltransferase 1 (*HPRT1*) were used as reference genes. Fold changes in level of gene expression was calculated by 2^^-ΔΔCT^ method.

Mean values ± SEM of two biological replicates were reported and statistical analysis was performed using the Mann-Whitney test by GraphPad Prism 6^®^ software.

## Results

### Perfusion Improves Cell Proliferation and Viability in 3D Culture

**Figure 2:**
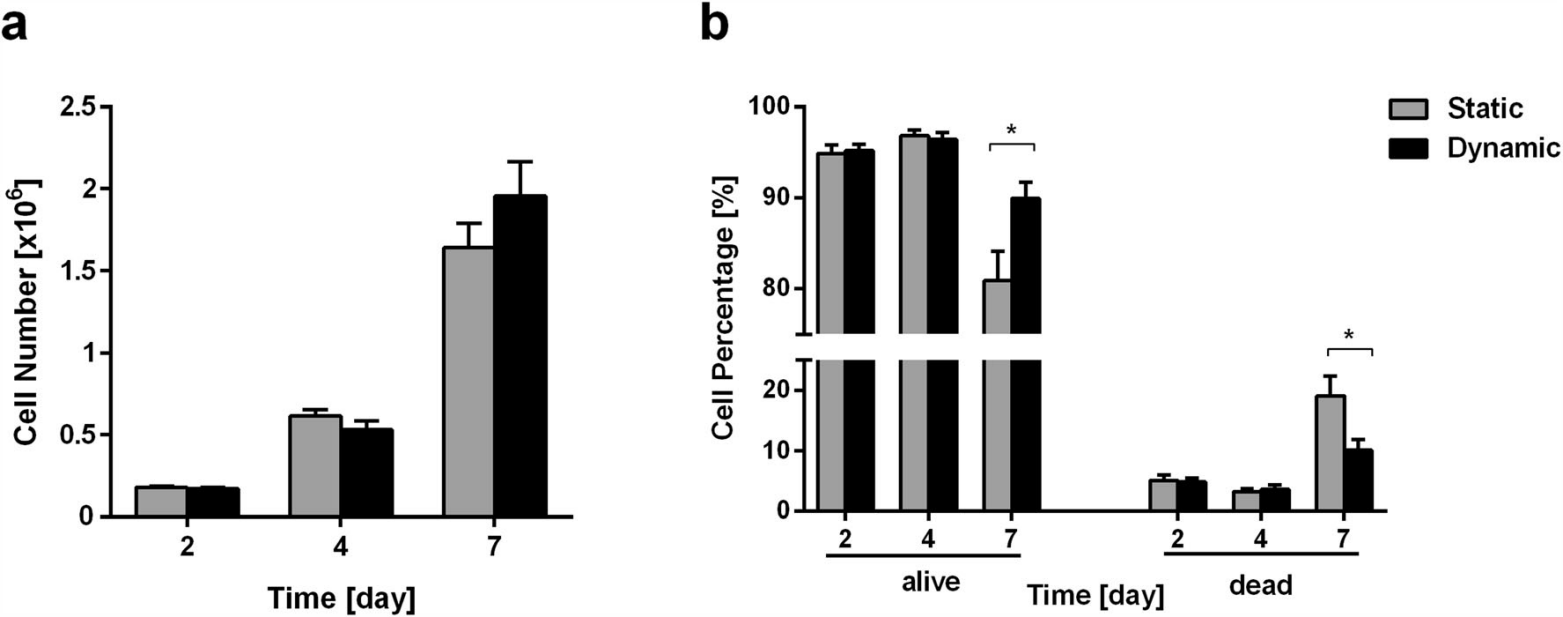
Comparison of cell viability and proliferation of MDA-MB-231 cells grown in 3D collagen scaffolds in static and dynamic cell culture conditions. (a) Cell counts at 2, 4 and 7 days of culture in static and dynamic conditions, (b) and dead cell percentages. Mean values and ± SEM of cell counts of at least 4–6 scaffolds per condition, cultured in at least 2 independent experiments. *p < 0.05.

To test the effect of perfusion on MDA-MB-231 cell proliferation and viability, we dissolved 3D collagen scaffolds with collagenase and counted live and dead cells at 2, 4 and 7 days of culture. At days 2 and 4 of culture, no significant differences in cell proliferation and viability were scored in static vs. dynamic condition. The number of live cells triplicates from day 2 to 4, from about 179k to 614k cells and 170k to 533k cells, in static vs. dynamic condition, respectively (Fig. 2a). After 7 days of culture the number of live cells triplicates again up to respectively 1.6 × 10^6^ and 2.0 × 10^6^ cells (Fig. 2a), with an increasing (20% more cells) trend of proliferation in dynamic vs. static condition. At the early time points no differences in cell viability were scored in terms of cell percentages when perfusion was applied: 94.9% (day 2) and 96.9% (day 4) of viable cells in static (5.1% and 3.1% of dead cells, respectively), and 95.2% (day 2) and 96.4% (day 4) of viable cells in dynamic condition (4.8% and 3.6% of dead cells, respectively). At the later time point, corresponding to 7 days of culture, a significant difference in dead cells was detected in static vs. dynamic condition (20.7% and 11.5%, respectively) (Fig. 2b).

**Figure 3:**
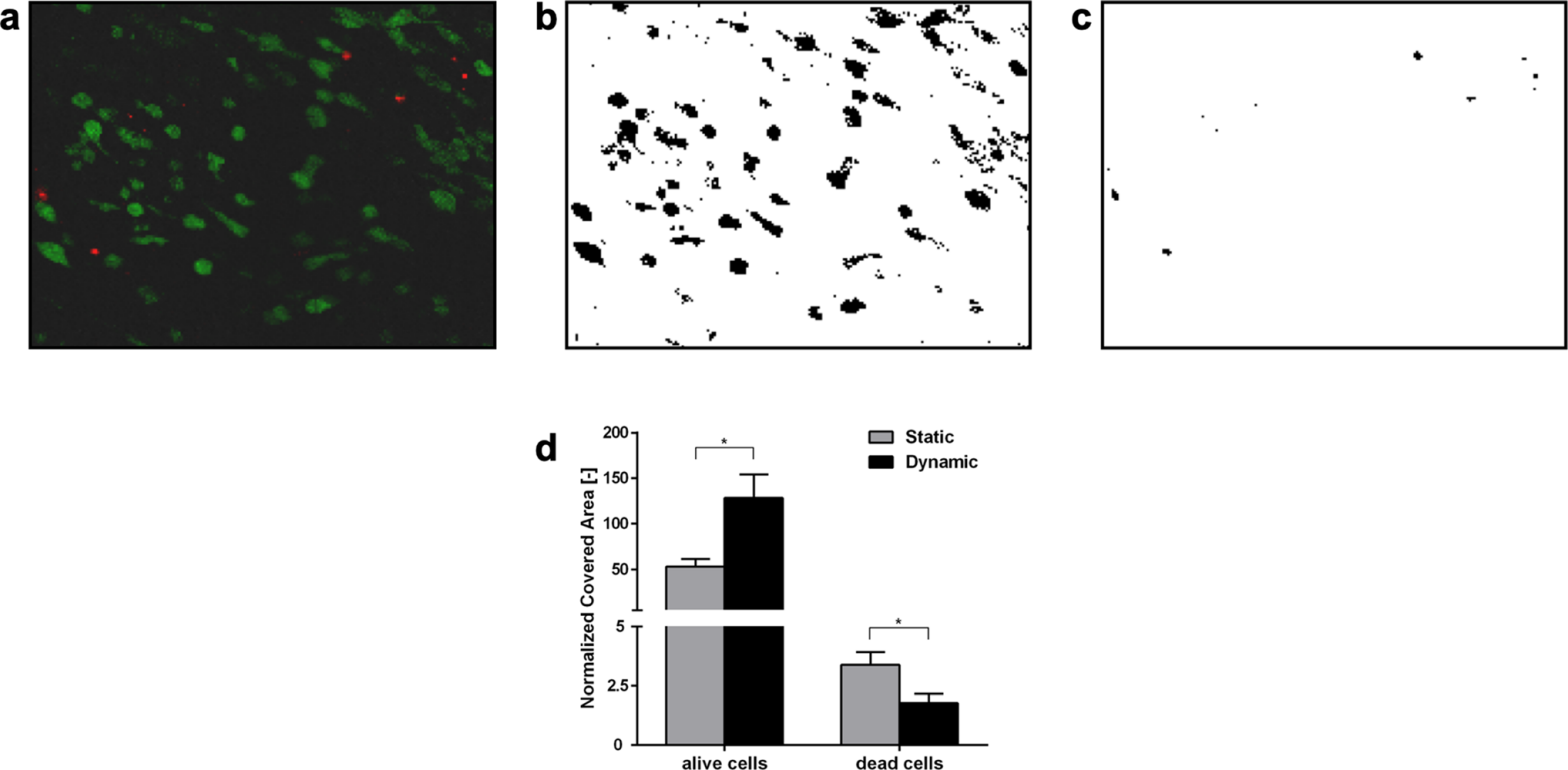
LIVE/DEAD^®^ cell viability assay. (a) Images acquired by fluorescent confocal microscopy (live cells in green, dead cells in red); (b, c) maximum entropy threshold-based image segmentation for live and dead cells respectively; (d) Quantitative analysis of the scaffold area covered by the live cells (green areas) and dead cells (red areas). Mean values and ± SD of at least 4 images for each condition and scaffold region. *p < 0.05.

To verify that hydrogel solubilization did not affect cell viability, LIVE/DEAD^®^ assay was performed on intact undissolved hydrogels after 7 days of culture in static and dynamic conditions. Live/dead images (Figs. 3a–3c) were analyzed to quantify (Fig. 3d) the segmented areas of the scaffold occupied by live and dead cells, respectively. The area covered by live cells was significantly higher (2.4 fold) in the dynamic condition and, consistently, the area covered by dead cells was significantly lower (0.52 fold). This quantification confirms cell counting data, showing that perfusion promotes cell viability.

### Perfusion Induces a More Homogeneous Distribution of Cells Within the Scaffold

The effect of perfusion on cell distribution within the scaffold was quantified through the analysis of images of DAPI/Phalloidin-stained cells cultured in static or dynamic conditions for 7 days.

**Figure 4:**
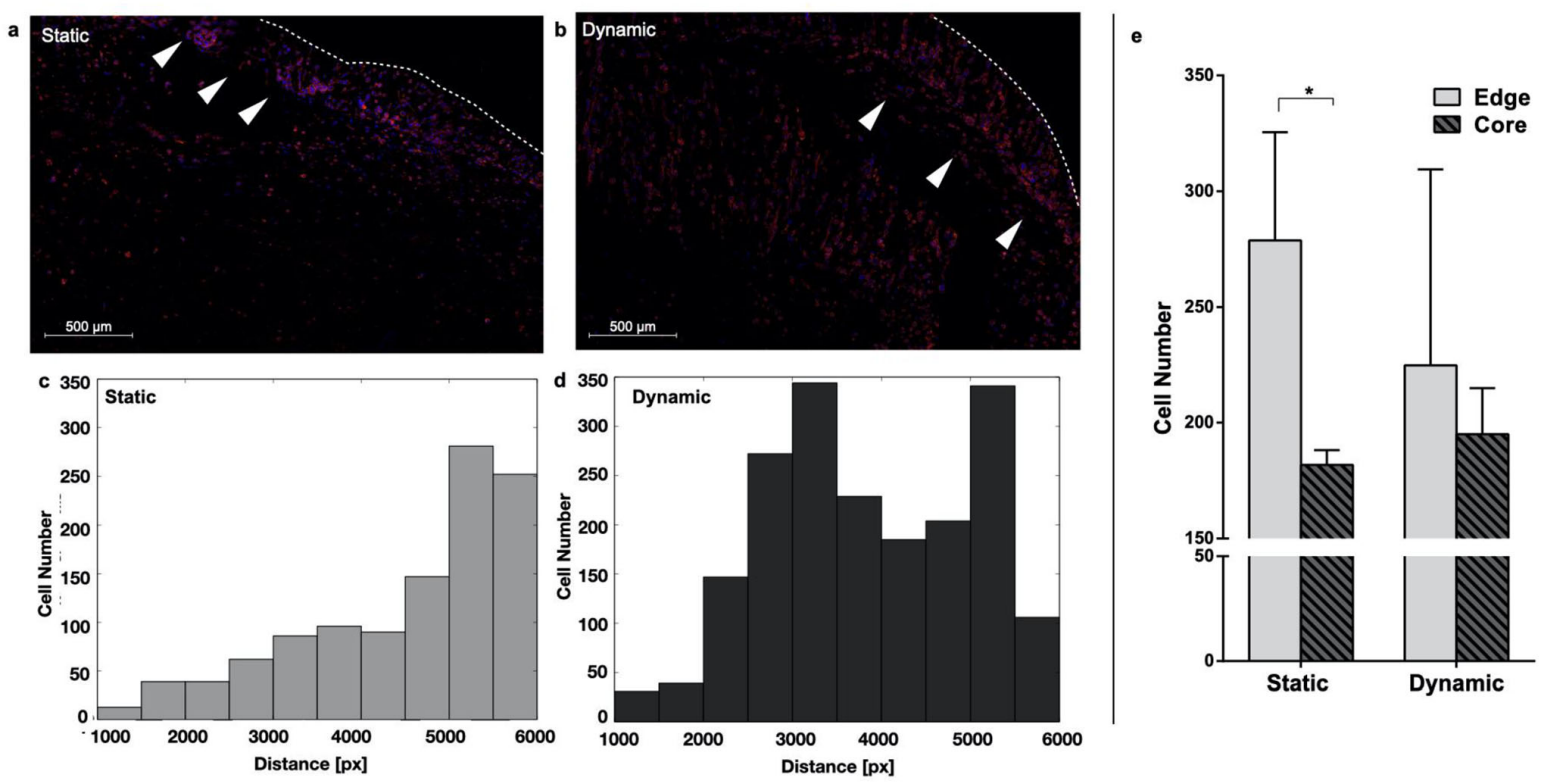
Analysis of cell distribution within the scaffold. Representative image of MDA-MB-231 cells stained with DAPI/Phalloidin after 7 days of culture in static (a) or dynamic (b) conditions. Arrowheads point to the outermost region of the scaffold while the dotted line identifies its edge. (c) Distribution of cells in (a) as a function of the distance from the scaffold center. (d) Same as in (c) but for the dynamic condition. (e) Quantification of the number of cells in each considered region of the scaffold (Edge/Core) in static vs. dynamic for the considered condition (3 to 7 images for each condition and scaffold region). *p < 0.05.

In static culture condition, cell distribution within the scaffold is more heterogeneous, with cells mainly clustered at the scaffold edge (Figs. 3a–3c); while when perfusion was applied, a more uniform cell distribution within the scaffold was scored (Figs. 4b–4d). This feature was quantified segmenting the DAPI signal from images of different regions (either edge or core) of the scaffold and counting the number of cells. The static culture condition was associated with a difference between the average number of cells on the border of the scaffold and in its center of about 30% (*p* value < 0.05), while the application of a perfusion flow reduces this difference to less than 20% (Fig. 4e).

### Perfusion Induces a Change in Cell Morphology

Phalloidin staining targeting actin filaments, was used to visualize the cell morphology. To this aim, Phalloidin signal was segmented as detailed in the methods section, and the cell eccentricity was computed. This quantity, that varies between 0 and 1, captures how much a given shape differs from a circle (eccentricity=0) and is equivalent to the aspect ratio used in Ref.^16^ to quantify actin fibers alignment.

**Figure 5:**
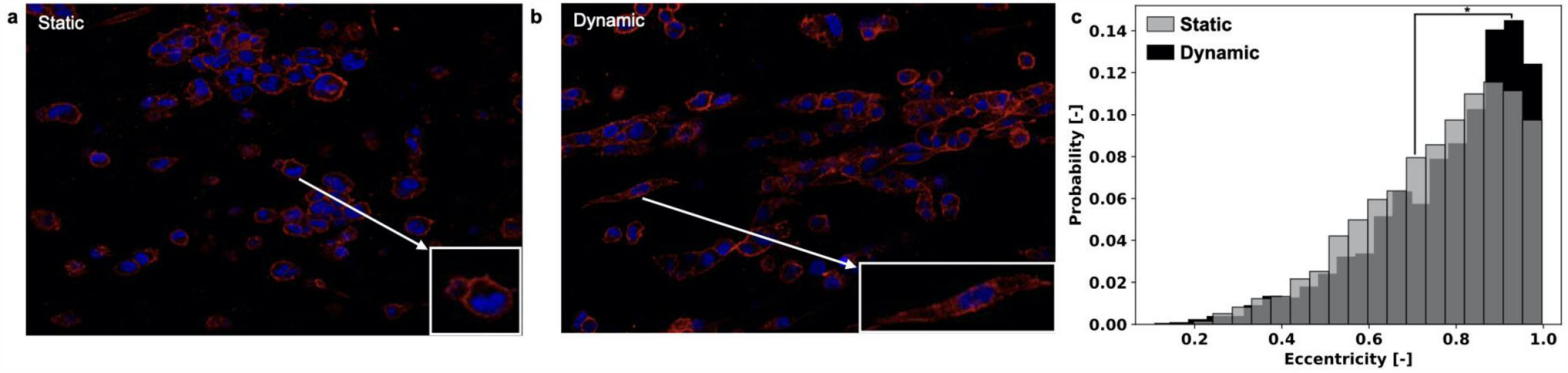
Analysis of cell shape. Representative image of MDA-MB-231 cells stained with DAPI/Phalloidin after 7 days of culturing in static (a) or dynamic (b) conditions. (c) Eccentricity distribution of cell shape recovered segmenting the Phalloidin signal as detailed in the methods section (at least 10 images/condition). *p < 0.05.

Figure 5 reports the result of this analysis. Perfusion induced marked changes in cell shape with rounded cells acquiring a spindle-like morphology (Figs. 5a–5b). This is confirmed by the corresponding eccentricity distributions (Fig. 5c). An analysis of these data recovered an increase in skewness of about 35% for the dynamic condition. This is associated with an increase in the probability of highly eccentric cells (Eccentricity >0.8).

### Perfusion Increases Cell Migration

Since confocal microscope images showed a different cell morphology and distribution in the scaffolds cultured in static vs. dynamic conditions, we further investigated cell behavior performing a scratch wound healing assay. MDA-MB-231 cells grown for 7 days in 3D collagen hydrogels in static or dynamic culture conditions were collected, after collagen digestion, and seeded in standard culture plates, where the wound healing assay was conducted capturing images at time 0, 2, 4, 6, 8, 24 h. Significant differences between cells previously grown in static vs. dynamic condition were scored after only 4 h. Perfusion induced an increase in migration ability of MDA-MB-231 cells. In detail, at 4 h the cell-free area representative of the wound was 62 and 78% in cells previously grown in dynamic and static culture, respectively. The effect of perfusion was more evident at later time points showing increased differences between the two cell culture conditions with cell-free area percentages of 54–73 (6 h), 46–70 (8 h) and 7–34 (24 h), respectively.

**Figure 6:**
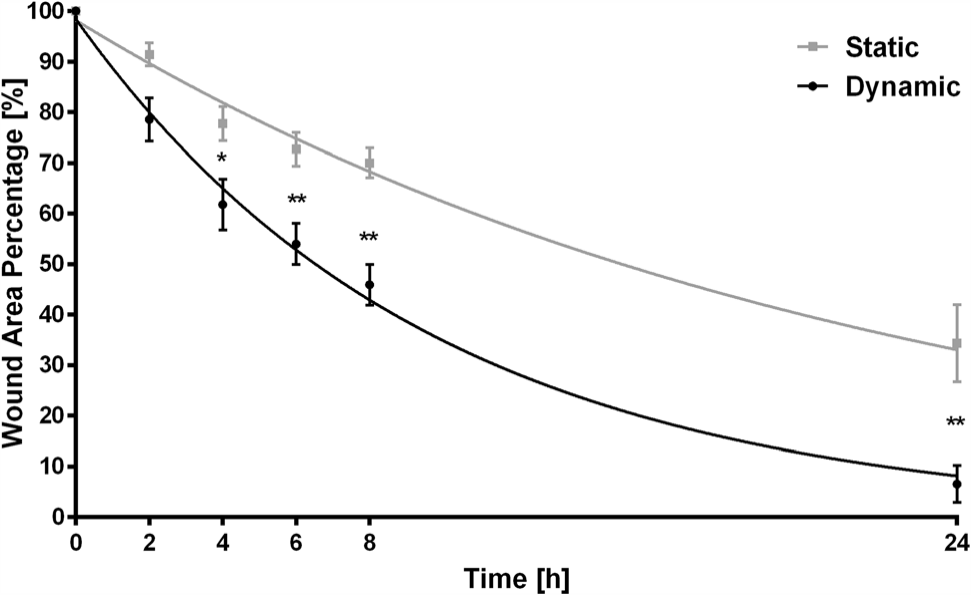
Scratch wound healing assay in MDA-MB-231 cells grown in 3D collagen scaffold for 7 days in static and dynamic conditions and seeded in standard tissue culture plates after collagen digestion. Images were captured at 0, 2, 4, 6, 8 and 24 h post-wound (at least 8 images per condition with cells obtained from at least 3 different scaffolds). Cell-free surface area was calculated over time and fitted with an exponential curve (grey squares/curve and black circles/curve for static and dynamic conditions, respectively). *p < 0.05; **p < 0.01.

The reduction in wound area was determined to follow an exponential dynamic (continuous lines in Fig. 6, *R*^2^ = 0.74 and 0.88 in static and dynamic conditions, respectively). This result points at a shift in the cells proliferative/migratory behavior with respect to standard culture in 2D, where the wound area linearly decreases, and is coherent with multiple sources highlighting how migration in 3D differs from that in 2D monolayers.^51^ Furthermore, this behavioral change is more pronounced in dynamic conditions where the time constant is over 2 times smaller when compared with the static one (*τ*_s_ = − 22.04 h [− 27.66 to − 12.70] and *τ*_d_ = − 9.64 h [− 11.57 to − 8.26]) and the time needed to fill 50% of the wound is halved $\left( {T^{ 50}_{\text{d}} = { 6}. 6 8 {\text{ h}},T^{ 50}_{\text{s}} = { 15}. 2 8 {\text{ h}}} \right)$.

### Perfusion Increases the Expression Level of Cell Invasion Markers

**Figure 7:**
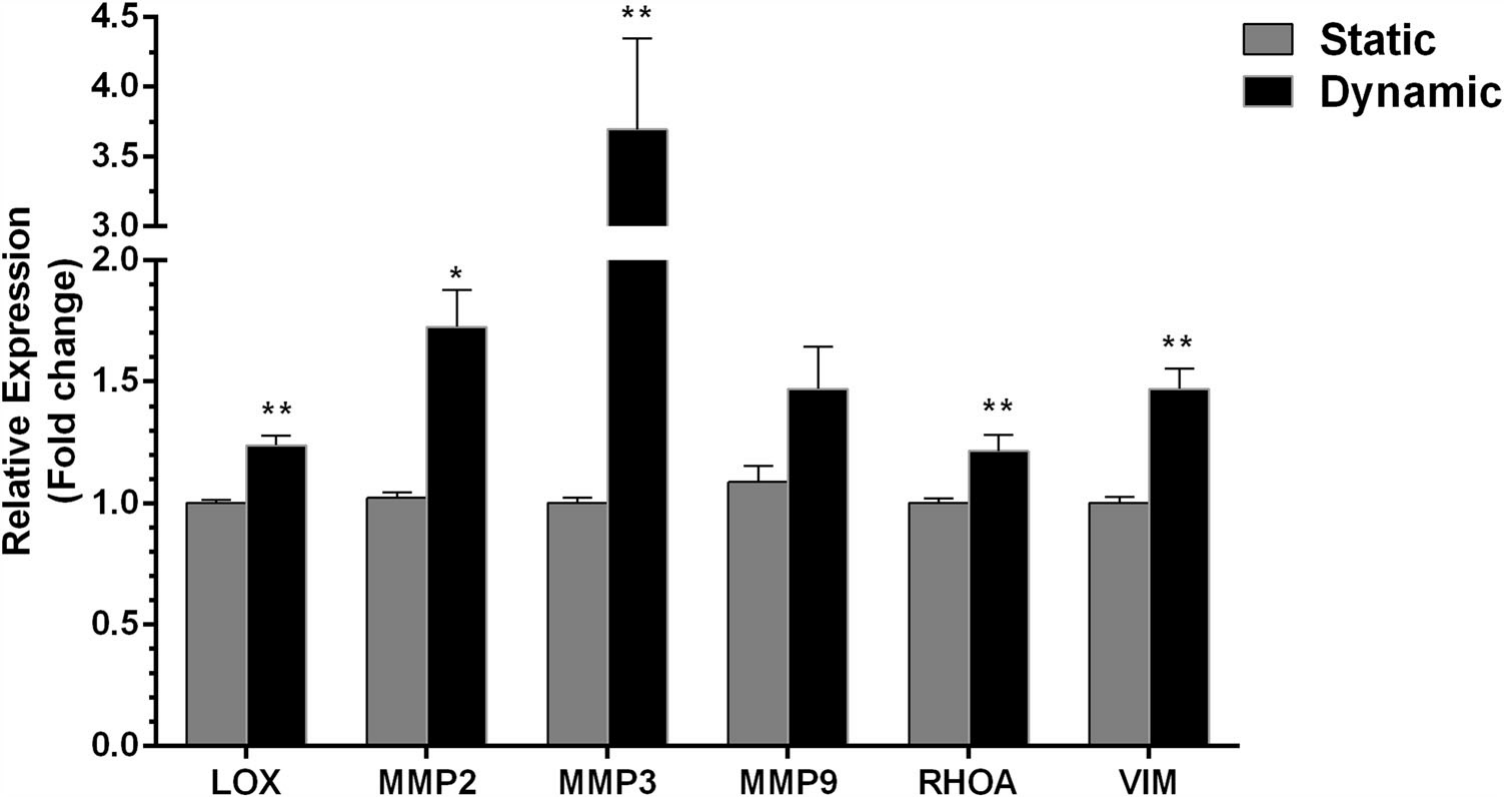
Gene expression analysis of MDA-MB-231 cells grown in 3D collagen scaffold (mRNA collected from 3 scaffolds per condition cultured in 2 independent experiments) for 4 days in static and dynamic conditions. The mRNA of invasion/migration related genes *LOX*, *MMP2-3-9*, *RHOA*, and *VIM* were analyzed by qPCR using *ACTB* and *HPRT1* as reference genes (2^−ΔΔCT^ method). Fold changes respect to static condition were calculated.*p < 0.05; **p < 0.01.

Morphological and behavioral changes detected at day 7 of MDA-MB-231 cell culture in collagen hydrogels under dynamic conditions, suggested to explore the expression status of genes implicated in invasive and migratory phenotype at an earlier time point corresponding to day 4 of culture. Figure 7 reports the results of this analysis. The mRNA expression level of genes playing a role in cell invasion and metastasis formation lysyl oxidase (*LOX*), three members of the matrix metalloproteinase group (*MMP2, MMP3* and *MMP9*), transforming protein RhoA (*RHOA*), and vimentin (*VIM*) were 1.24, 1.73, 3.69, 1.47, 1.22 and 1.47 fold increased, respectively, in dynamic vs. static condition (*p* values < 0.05, except for *MMP9*), suggesting that perfusion was associated with a more aggressive phenotype characterized by enhanced migratory ability.

## Discussion

It is nowadays recognized that 3D culture is fundamental to study and more realistically reproduce biological or pathological *in vivo* cell behaviors.^18^ This is particularly important when studying cancer features, such as invasion, motility and metastasis formation, considering the essential role of microenvironment in regulating tumor progression. For breast cancer, in particular, many examples in the literature highlight that 3D culture conditions induce a more aggressive and resistant phenotype, both in presence or absence of mechanical dynamic stimulation.^8^,^40^

In this study, we couple 3D cell culture in collagen hydrogels and perfusion to study the behavior of an aggressive breast cancer cell line MDA-MB-231 in order to investigate how the phenotype of this 3D *in vitro* model could be affected by dynamic culture conditions.

In accordance with other recent evidence collected in different models of 3D-perfused cancer cell culture,^2^,^3^,^22^,^23^,^40^ we show that cell proliferation and viability were improved by perfusion at prolonged days of culture, when it is likely that the observed increased number of cells created competition for nutrients and oxygen (Figs. 2, 3). Although this result suggests that diffusion of nutrients and oxygen, together with removal of waste, contribute to enhance cell culture conditions in our bioreactor system,^43^ the improvement in perfusion-driven cell proliferation might appear less pronounced (Fig. 2a) than it could be expected. This issue deserves to be further investigated in future experiments with higher cell seeding densities in order to more closely reproduce a cell-dense neoplastic environment. In the present experiment we chose to start with reduced initial cell densities to have a chance to study other cell parameters, such as cell distribution within a widely colonizable environment.

While a hypoxic core occurring in 3D culturing systems in standard static condition results in a non-uniform cell distribution within the 3D structure and could affect cell viability in inner regions,^13^,^19^,^33^ in our system, perfusion also determines a more homogenous distribution of cells, as shown in Fig. 4. Additionally, dynamic cell culture conditions were associated with different cell features and behavior with respect to their counterparts grown in the static 3D culture. Perfused MDA-MB-231 cells exhibited a more elongated shape (Fig. 5), which usually characterizes cells with higher motility capacity. This result was confirmed by their increased ability to repopulate the gap in a scratch wound healing assay (Fig. 6). Moreover, a gene expression analysis showed that dynamic culture conditions are able to modify the molecular phenotype of the cells (Fig. 7), in accordance with other studies where cells were cultured in a 3D setting.^20^,^21^

*LOX*, *MMP2*, *MMP3*, *MMP9*, *RHOA*, *VIM* gene panel expression monitoring was considered because they are correlated with a more aggressive and invasive phenotype in breast cancer cells. Since we aimed to evaluate how the perfusion system can support, in a long-term culture, the growth and the invasive migratory behavior of MDA-MB-231 cultured in an *in vitro* 3D model, we hypothesized that modulation of these selected genes would represent a valid indicator.

In particular, proteins encoded by the above genes play a key role in promoting cancer’s cell invasive phenotype: MMP2, MM3, MMP9 are metalloproteinase capable of degrading the extracellular matrix, a fundamental step for cell migration^52^; LOX is a hypoxia induced-ECM remodeler which was found strictly correlated with an invasive and metastatic phenotype in triple-negative breast cancer cells^32^; VIM is correlated to the biological process of epithelial-mesenchymal transition, and is considered a marker of acquisition of a more mesenchymal and aggressive phenotype, essential for cancer cell spread in a biological tissue^41^; finally RHOA belongs to RHO GTPase family, key players in regulating tumor cell migration since mediating actin stress fiber formation, and was found hyperactivated in breast cancer patients associated with distant metastasis.^6^,^30^ All together, the gene panel expression upregulation in our 3D model highlights that MDA-MB-231 cells show a more invasive and aggressive molecular phenotype when cultured under dynamic conditions.

These results confirm the need of experimental settings that more reliably reproduce tumor cell behavior to offer *in vitro* models able to provide more realistic results, especially for drug screening studies. However, the transition from standard 2D to perfused 3D culture strategies opens new research challenges, since most of the biological assays and analysis methods are optimized for cells grown in monolayer and lack of quantitatively measurable information, especially when based on microscope images.

In this study, we show that image analysis techniques could be employed to address these limitations, effectively quantifying the results of traditional *in-vitro* assays, while also providing additional information which would be otherwise undetectable. While other examples of this approach are available in the literature,^12^,^14^,^49^,^50^ its application to 3D scaffolds is still limited.^1^,^7^,^8^,^35^,^40^,^45^ To demonstrate the applicability and usefulness of quantitative image analysis methods for the study of scaffold-based 3D cell cultures, we have shown that cell viability evaluated on live/dead assay images^45^ provides comparable results to cell counting following scaffold digestion. Additionally eccentricity analysis^24^ was determined to be able to effectively quantify cell morphology, which is generally studied only qualitatively, and the measurement of cell invasiveness/aggressiveness *via* processing of scratch wound healing assay images^15^ yielded results coherent with gene expression analysis. The use of image analysis, furthermore, preserves cell localization within the scaffold, allowing to study how local microenvironment affects cell behavior.

In conclusion, combining 3D cell culture with a perfusion bioreactor system represents an effective compromise between a realistic representation of *in-vivo* behavior^8^,^28^ and a feasible experimental setting, that could be fundamental for the study of specific cancer cell biological features.
